# How do food safety technical working groups within a One Health framework work? Experiences from Vietnam and Ethiopia

**DOI:** 10.1186/s42522-024-00110-y

**Published:** 2024-09-02

**Authors:** Steven Lâm, Sinh Dang-Xuan, Meseret Bekele, Kebede Amenu, Silvia Alonso, Fred Unger, Hung Nguyen-Viet

**Affiliations:** 1 International Livestock Research Institute, Naivasha Road, P.O. BOX 30709-00100, Nairobi, Kenya; 2 International Livestock Research Institute, Hanoi, Vietnam; 3 International Livestock Research Institute, Addis Ababa, Ethiopia; 4 Ministry of Agriculture, Addis Ababa, Ethiopia; 5 College of Veterinary Medicine and Agriculture, Addis Ababa University, Bishoftu, Ethiopia

**Keywords:** Food safety, Technical working group, One Health, Collaboration, Success factors, Vietnam, Ethiopia

## Abstract

**Background:**

Persistent challenges of fragmented, food safety management in low- and middle-income countries underscore the need for more robustly coordinated mechanisms. National food safety technical working groups, operating under a One Health framework, offer potential in streamlining coordination efforts to effectively address these challenges. However, more clarity regarding their formation and functioning is important for understanding how to best establish and support such groups. The aim of this study is to systematically document the development process of established groups in Vietnam and Ethiopia.

**Methods:**

We assess the process used to establish and support the technical working groups against six critical success factors for multisectoral collaboration: drive change, define, design, realise, relate, and capture success. To do so, we review meeting minutes, Terms of Reference, and other related publications.

**Results:**

The analysis underscores the importance of financial and technical support by development partners in initiating working groups while also highlighting the challenge posed by the absence of legal frameworks to secure government commitment. Embedding the technical working groups within existing government structures – such as One Health platforms – from the outset could help to ensure the active participation and sustainability of such groups.

**Conclusion:**

Both Vietnam and Ethiopia have established operational and institutionalized technical working groups to bolster national food safety efforts under a One Health framework. The approaches employed in these countries could serve as valuable models for others seeking to establish comparable multisectoral collaborative mechanisms to address emerging health risks.

## Background

Global and regional strategies for food safety underscore the need for multisectoral coordination mechanisms to address emerging health risks [1, 2]. However, implementing a multisectoral approach is challenging, particularly within low- and middle-income countries (LMICs) where foodborne diseases are becoming increasingly prevalent [3]. Many of these outbreaks are linked to the informal food sector, which can include small-scale producers, street vendors, and home-based food businesses that operate outside of formal regulatory frameworks [4]. Encouraging informal value chain actors to adopt new practices or comply with regulations may face resistance if these changes are seen as disruptive to routines or difficult to apply in practice.

To strengthen multisectoral coordination mechanisms, it is essential to develop a structure – defined in legislation – for the oversight and operation of the coordination mechanism. As many countries have existing national structures – such as One Health platforms – situating food safety coordination within these structures offers an entry point as it optimizes the use of available resources [5]. One Health recognizes that the health of humans, animals, and the environment are intertwined, and, as such, advocates for addressing health risks through a multisectoral approach, involving experts from fields such as human health, veterinary medicine, food safety, and environmental science [6].

The current risks to food safety – forecasted to intensify in a changing climate – make it imperative to understand and establish robust stakeholder coordination mechanisms in LMICs [7]. Food safety technical working groups (FSTWG) can play a key role in fostering this coordination but how they work and how they can be embedded within existing structures such as One Health platforms remains understudied [8]. As such, the aim of this study was to systematically document and learn from the establishment process of FSTWGs. This work draws from the experiences of Vietnam and Ethiopia, where FSTWGs were established within the national One Health structures in 2023.

### About food safety technical working groups

Creating FSTWGs can be an efficient approach to coordinate food safety efforts at the national level. These groups offer a forum that brings together experts and stakeholders to voluntarily collaborate on shared objectives. By their nature, technical groups can respond quickly to food safety issues, leveraging the collective expertise of their members. Furthermore, in LMICs, where there may be a need for adapting international best practices, these groups can play a role in facilitating the transfer of knowledge and the localization of international practice. These groups also often engage in advocacy efforts to influence policies related to food safety at the national and international levels [9].

**Fig. 1:**
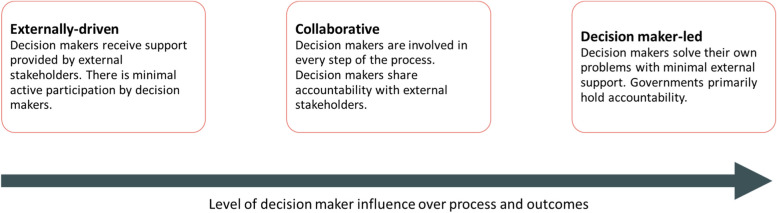
Typology of food safety technical working groups (adapted from [11])

FSTWGs across countries can share common goals but the composition and priorities can vary. In high-income countries like the U.S., where regulatory frameworks are already well-established, governmental bodies such as the Department of Health and Human Services may form interagency working groups ad hoc to align efforts and address domestic food safety concerns [10]. Meanwhile, within LMICs, FSTWGs may be led by international organizations, national organizations, and donor agencies, which offer expertise to governments. While development partners can provide support, ownership must transition to governments and other decision making partners for long-term success [8]. We present a typology of possible FSTWGs, defined by their degree of decision maker ownership (Fig. 1).

## Methods

### Context: CGIAR support for food safety technical working groups

CGIAR, a global research partnership committed to reshaping food systems under a climate crisis, oversees 32 initiatives funded from 2022 to 2024. Two initiatives – the One Health Initiative, addressing zoonotic and foodborne diseases, and antimicrobial resistance in food systems of LMICs [12], and the Resilient Cities Initiative, targeting urban food system challenges in the Global South and enhancing city resilience amid rapid urbanization – support FSTWGs in Vietnam and Ethiopia [13]. These initiatives offer both technical support (e.g. organizing gatherings) and financial assistance (e.g. covering meeting expenses) to the FSTWGs.

Based on 2020 data gauging countries’ strengths in food safety coordination, many LMICs rated themselves as “no capacity” or “limited capacity” [4, 14]. In Vietnam and Ethiopia, where CGIAR maintains ongoing partnerships with government bodies, requests for coordination support have shaped initiative efforts to strengthen (in the context of Vietnam) or establish (in the context of Ethiopia) FSTWGs. Considering that both Vietnam and Ethiopia are middle- and low-income nations, respectively, characterized by diverse regulatory systems, sharing experiences of the FSTWG development process can facilitate the development of insights into multisectoral coordination toward safe food. These regions exhibit commonalities, such as fragmented food safety measures and overlapping responsibilities, serving as the foundation to evaluate both the generalizability and context-specific nature of coordination initiatives.

### Approach

To capture the process of FSTWG development, we used a hybrid deductive-inductive approach to thematic analysis [15]. We first identified meeting minutes and other relevant publications through websites of FSTWGs; we also drew on expertise of our authorship team, many of whom played key roles in establishing FSTWGs within their respective countries. Then, we deductively coded segments of text using an analytical tool that we developed. In this tool, we defined the following three themes: context, process, and opportunities/challenges. Further themes were inductively developed to accommodate data that could not be coded into one of the predetermined themes, such as commonalities and differences across the two FSTWGs.

A British Medical Journal series offered strategies on how best to work across sectors to achieve better health and sustainable development, drawing on 12 country case studies of multisectoral collaboration [16]. A synthesis of these studies identified six success factors for multisectoral collaboration: drive change, define, design, realise, relate, and capture success (Table 1) [17]. We selected these factors as a framework to help analyze and tell the story of FSTWGs given their comprehensiveness in covering the different steps of multisectoral collaboration and relevance to LMICs. Following coding, we organized thematic insights under these factors.

**Table 1:** Enabling factors for effective multisectoral collaboration [17]

Enabling factor	Description
**Drive change**	Assess whether the desired change is best achieved by multisectoral collaboration. Drive forward collaboration by mobilising a critical mass of policy and public attention
**Define**	Frame the problem strategically and holistically so that all sectors and stakeholders can see the benefits of collaboration and contribution to the public good
**Design**	Create solutions that are relevant to each context, build on existing mechanisms, and leverage the strengths of diverse sectors for collective impact
**Relate**	Ensure resources for multisectoral collaboration mechanisms, including open communication and deliberation on evidence, norms, and innovation
**Realise**	Learn by doing and adapt with regular feedback. Remain open to redefining and redesigning the collaboration to ensure relevance, effectiveness, and responsiveness to change
**Capture success**	Agree early on markers of success. Use qualitative and quantitative methods to monitor results regularly. Learn from failures and successes to inform action and sustain gains

## Results

### Overview of FSTWGs in Vietnam and Ethiopia

**Fig. 2:**
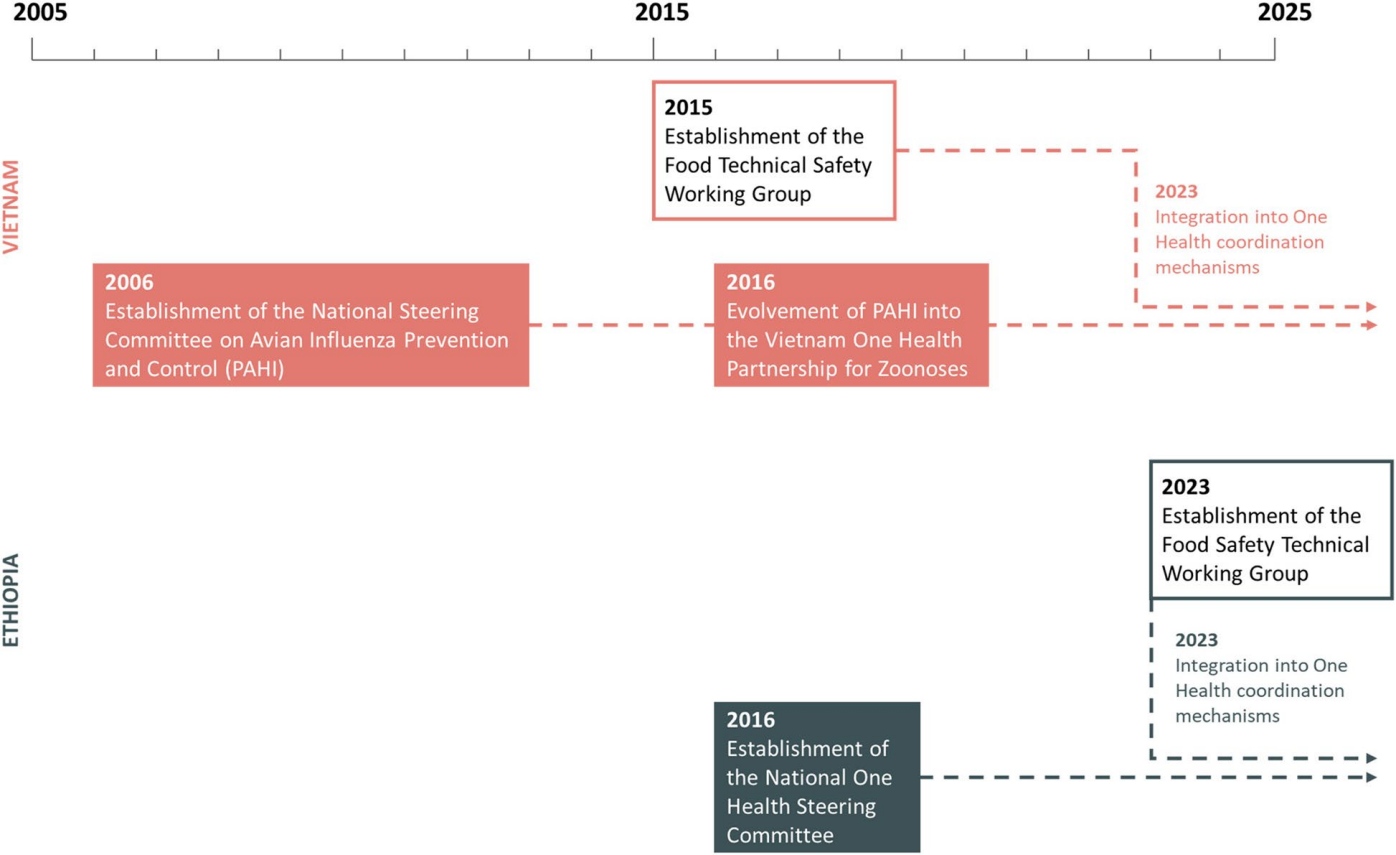
Timeframe of the development of food safety technical working groups and One Health coordination mechanisms in Vietnam and Ethiopia

FSTWGs in Vietnam and Ethiopia shared the objective of providing a forum for stakeholders to share information, collaborate, and develop policy advice in matters related to food safety at the national level (Table 2). These FSTWGs have different set-ups and organizational structures, which arose from different starting points. In Vietnam, the FSTWG has been involved in shaping national food safety responses since its inception in 2015, largely supported by the International Livestock Research Institute (ILRI) and development partners (Fig. 2). In 2023, it was integrated into the national One Health mechanism – the Vietnam One Health Partnership (OHP). In Ethiopia, the recognition of the need for the FSTWG arose during the establishment of the country’s One Health mechanism – the National One Health Steering Committee (NOHSC) – in 2016. However, the FSTWG was not formally initiated until 2023. Unlike the case of Vietnam, Ethiopia’s FSTWG was integrated into the country’s One Health mechanism right from its inception.

**Table 2:** Structure of food safety technical working groups in Vietnam and Ethiopia

Characteristics	Vietnam	Ethiopia
Purpose	Shared objective of providing a forum for experts and food safety stakeholders to share information, collaborate, and develop policy advice on food safety at the national level	
Scope	Broad in scope, potentially addressing multiple stages of the food supply chain (e.g. production, processing, distribution, consumption) as well as multiple hazards (e.g. biological, chemical, physical risks) without focusing on a particular type	
Core activities	- Scientific (e.g. compile scientific evidence on the food safety risks, develop risk assessment tools) - Policy (e.g. develop recommendations to update existing legislations, compile examples of successful models) - Communication (e.g. develop plans to promote good practices, organize awareness-raising events)	- Coordination (e.g. create opportunities for information sharing, maintain a database of on-going initiatives) - Policy (e.g. develop recommendations to update existing legislations, advocate for risk-based approaches to food safety) - Communication (e.g. develop plans to promote good practices, participate in awareness-raising events)
Set-up and operations	- Established in 2015 - Integrated under the Vietnam One Health Partnership later in 2023 - Meetings held quarterly - All members participate in meetings and contribute to activities	- Established in 2023 - Integrated under the National One Health Steering Committee upon establishment - Meetings held quarterly - All members participate in meetings and contribute to activities
Governance	- Chair and co-chair, roles filled by government representatives or development partners on a rotating basis every two years, coordinate meetings - Secretariat, filled by a government representative on a rotating basis every two years, manages administrative tasks	- Chair and co-chair, roles filled by government representatives on a rotating basis every year, coordinate meetings - Secretariat, filled by a government representative or development partner on a rotating basis every year, manages administrative tasks
Government representatives initially involved	- Ministry of Health - Ministry of Agriculture and Rural Development - Ministry of Industry and Trade - Office of the Government	- Ministry of Health - Ministry of Agriculture - Ministry of Trade and Regional Integration - Environmental Protection Authority - Ethiopian Wildlife Conservation Authority
Development partners initially involved	- World Bank - Canadian Embassy to Vietnam - Embassy of New Zealand - Food and Agriculture Organization of the United Nations - World Health Organization - Japan International Development Agency - Asian Development Bank - International Livestock Research Institute	- The Ohio State University-Global One Health Initiative - World Health Organization - Food and Agricultural Organization - United Kingdom Health Security Agency - United States Agency for International Development - World Food Programme - International Livestock Research Institute - Johns Hopkins Center for Communication Programs - Centers for Disease Control and Prevention

### Overview of One Health platforms in Vietnam and Ethiopia

FSTWGs were developed to help One Health platforms achieve their objectives related to national food safety. Platforms are institutionalized structures to promote formal, inter-ministerial coordination. Nested under platforms, FSTWGs play essential advisory and operational functions for platforms to inform their decision-making relating to policies, implementation of One Health plans, and emerging issues (Table 3).

**Table 3:** Structure of One Health platforms in Vietnam and Ethiopia

Characteristics	Vietnam	Ethiopia
Purpose	Shared objective of addressing health issues at the interface of human, animal, and environmental health through multisectoral collaboration using a One Health approach	
Scope	Addresses a range of issues including disease surveillance, zoonotic disease control, antimicrobial resistance, food safety, and environmental health	
Core activities	Coordination across sectors, policy and strategy development, surveillance and response to health threats, capacity building, advocacy, research, and resource mobilization	
Governance	- Chair and co-chairs, roles filled by government representatives, coordinates activities between ministries, develops and implement national One Health plans, and establishes technical working groups (e.g. for zoonoses, antimicrobial resistance, food safety, etc.) - Secretariat, role filled by a government representative, manages administrative tasks - Technical working groups, nested under One Health platforms, comprise experts from relevant fields and serve advisory and operational functions for platforms	
Setup and operations	Established in 2016 by stakeholders including 27 ministries and development partners in Vietnam, which expanded to 31 stakeholders since the launch of the new partnership framework in 2021	Established in 2016 by stakeholders including eight ministries and development partners in Ethiopia

### Analyzing FSTWG success factors in Vietnam

#### Drive change

Repeated episodes of adulterated and unsafe food practices led the public to demand urgent action [18]. The FSTWG was created at the request of Deputy Prime Minister Vu Duc Dam during a meeting convened under his auspices in June 2015 to enhance public health and public trust [19]. The FSTWG brought together government agencies, development partners, and researchers for policy dialogue and discussions. The overall goal was to contribute to improved national food safety and increased competitiveness of food products, recognizing that accomplishment of this goal is best achieved by multisectoral collaborations.

#### Define

Once a decision to engage in multisectoral collaboration was taken, attention was paid to defining collective objectives, which included:

1. To serve as a forum for food safety stakeholders including members from academia, the development sector, and the private sector to share their work on food safety from Vietnam and other countries with relevance to Vietnam. The FSTWG in Vietnam takes a broader, higher-level perspective rather than focusing on a specific commodity or risk.
2. To promote specific policy issues for consideration by the government and to serve as a resource to consult with on draft policies, regulations, and other high-level documents.
3. To harmonize and synergize donor efforts in terms of financial, technical, and capacity-building support in food safety.

#### Design

Because the FSTWG was a completely new coordination mechanism, processes were established to ensure the group was effective. For example, a Terms of Reference (TOR) outlined the objectives of the FSTWG, the expected and optional contribution of members, and the frequency and duration of meetings. By joining the FSTWG, member organizations agreed to commit the time of a senior representative and technical staff to actively participate in the meetings and contribute to food safety improvement efforts. Of note, the FSTWG was not established under an official decision – a formal resolution made by a governmental authority – which limits the extent to which government actors could engage.

#### Relate

Mechanisms were developed to support group operations, including principles for open communication and distribution of effort. Group members volunteered their time to participate in and/or chair meetings. In Vietnam since 2015, the group has been chaired by the Food and Agriculture Organization of the United Nations (FAO), the World Bank, and ILRI, none of which are government actors. A core group planned the quarterly meetings, with financial resources provided by ILRI when needed (e.g. to cover in-person meeting expenses).

#### Realise

Regular reflection enabled the FSTWG to refine its approach when initial plans failed to achieve desired results. For example, engaging all members in planning was difficult to manage. Having a core group that consulted the wider group made it easier to plan meetings and activities. Additionally, recognizing the contributions of government actors were limited, which reduced the ability to influence high-level actions, the group sought to increase government engagement. The OHP – established under an official decision in 2016 – created working groups to achieve objectives relating to future pandemics, antimicrobial resistance, and policy advocacy. Rather than create a new group for food safety, the FSTWG proposed for it to be adopted as a working group for food safety under the OHP, which was approved in 2023 [20]. Until 2023, the FSTWG was largely a platform for exchange between development partners and researchers.

The OHP was eager to assume responsibility for the FSTWG due to its established track record. Moreover, food safety was a key component of the OHP’s strategic plan [21]. While the group's goals and activities will largely remain consistent, this restructuring has the potential to enhance the engagement of government entities. The OHP secretariat, situated at the Ministry of Agriculture and Rural Development, will serve as the host for the group’s secretariat and provide essential support including managing meeting logistics, creating meeting summaries, and maintaining communication channels. Group activities will be integrated into the OHP's action plan and budgetary framework.

#### Capture success

The group defined success as informing food safety decision making. Through scientific, policy, and communication activities, the group contributed to new outputs and relationships that over time could inform such decision-making. Notable examples include:

- Contributing technical insights to a World Bank-led food safety report in Vietnam: The World Bank released a report on food safety risk management in Vietnam, strengthened by the technical inputs by FSTWG members [19].
- Facilitating a global food safety expedition in Vietnam: FSTWG members together with the OHP organized a food safety expedition in April 2023 [22]. A total of 40 delegates from 15 countries learned about One Health food safety interventions in Thai Nguyen Province, a field site for One Health research and practice.
- Enriching a National Action Plan: FSTWG members helped to shape Vietnam's National Action Plan for Food Systems Transformation, particularly action track one focused on food security [23]. While the initial emphasis was on value chains and nutrition, the group advocated for food safety, contributing to its integration.
- Informing the development of a national food safety risk assessment centre: As pioneers in advancing food safety risk assessments, FSTWG members were invited to a workshop in Vietnam in December 2023. This workshop, coordinated by the National Institute for Food Control and the Ministry of Health, focused on effective funding and operation of a new national centre focused on food safety risk assessment.
- Enhancing capacity and publishing in the field of food safety risk assessment: FSTWG members actively participated in developing the food safety risk assessment curriculum, conducting training sessions, engaging in research on food safety, and disseminating scientific findings in this domain [24].
- Communicating food safety: FSTWG members regularly worked with media and journalists from public health, animal health, and agricultural sectors on food safety [25].
- Expanding membership: The FSTWG shifted from an initial research-focused group of 10 members to a multisectoral body of 60 members integrated into the national One Health framework.

### Analyzing FSTWG success factors in Ethiopia

#### Drive change

In 2016, four key Ethiopian Ministries joined together to establish the NOHSC with the support of the government of Ethiopia and development partners [26]. Currently, the NOHSC hosts several specific One Health technical working groups, including rabies, anthrax, brucellosis, emerging pandemic threats, antimicrobial resistance, and One Health communications, to foster inter-ministry collaborations on One Health-related initiatives. These groups received technical and financial assistance from development partners.

Of note, establishing a working group on food safety was part of NOHSC’s annual plans since its inception, but it was only until 2023 that support was received from CGIAR initiatives to create the FSTWG. The initiatives brought not only financial resources but also experiences supporting FSTWGs in Vietnam. In the short term, the initiatives aim to establish a framework so that, within three years, the FSTWG can operate independently under NOHSC's leadership, without further financial support from the initiatives. The long-term goal is recognition of the FSTWG as a technical body for providing expert advice on food safety matters. With knowledge of Ethiopia's One Health structure, the initiative approached key individuals of NOHSC with the proposal to establish a FSTWG, which was approved by NOHSC.

Highlighting the significance of NOHSC's interest in having a FSTWG, it is crucial to note that this effort coincided with heightened attention to food safety. The African Union developed a food safety strategy for the continent spanning 2022 to 2036 [2]. Furthermore, Ethiopia developed several strategies including the National Food Safety Strategy [27], the National Pathway for Food System Transformation [28] and the National Food Safety and Quality Strategy for Primary Agricultural Produce [29], all of which outline commitments to strengthening national food safety coordination. In Ethiopia, as the strategies were being developed, there was an absence of a forum for constructive dialogue and information exchange regarding who was engaged in which activities and who could offer support. Establishing an FSTWG was envisioned to fill this gap and facilitate such discussions. The enthusiastic reception of this idea can be attributed to the conducive environment and the demand for food safety support within Ethiopia.

#### Define

Similar to Vietnam’s FSTWG, the objective of the FSTWG in Ethiopia was to provide a forum for stakeholders in Ethiopia to share information, foster collaboration, and formulate policy advice on matters related to food safety in the country. The process began with the team taking the lead in drafting the TOR, which involved multiple rounds of revisions with NOHSC chairs. The TOR was adapted from those used in Vietnam to suit the Ethiopian context, as well as drawing insights from TORs used by other working groups of the NOHSC. Crucially, the design process benefited from the involvement of a team member who previously worked with the NOHSC, offering valuable insights into the process. Subsequently, the drafted TOR was presented to and received approval from the broader NOHSC group.

#### Design

By joining the FSTWG, member organizations commit to attending regular and ad hoc meetings, proposing issues to be addressed, and planning and implementing activities. It is worth noting the engagement of ministries is based on a Memorandum of Understanding. Initially, ministry personnel worked on NOHSC activities when they had spare time. However, in 2023, the NOHSC underwent institutionalization, resulting in a legal framework that facilitated collaborative endeavours among ministries. This institutionalization also entailed the allocation of a specific budget, the appointment of personnel, and the establishment of reporting mechanisms, all geared towards enhancing government accountability and amplifying the potential for impactful outcomes.

#### Relate

The initiatives leveraged existing relationships with NOHSC members, facilitated by the initiative members’ presence in various other technical working groups under NOHSC (e.g. zoonoses). Mechanisms are in place to ensure ongoing communication, with meetings convened every three months. Leadership roles, including chairs and secretariat, will undergo an annual rotation. The chairing positions are always held by the government, while the secretary positions are open to both the government and development partners.

#### Realise

While the FSTWG is a recent development, the team has actively applied lessons learned from past experiences. Leveraging insights gained from involvement in Vietnam, the team integrated the Ethiopian FSTWG into the existing national One Health mechanisms from the outset. This could mitigate the risk of a decline in government participation over time. Additionally, during the launch of the FSTWG under the NOHSC in October 2023, concerns arose regarding the absence of representation from specific key food safety stakeholder groups [30]. This situation reflects the "storming" stage of team development, where members begin to feel more at ease expressing dissenting viewpoints [31]. The team clarified that this inaugural meeting marked the starting point of this collaboration; organizations are welcome to join at any time and contribute to the collective effort. A key lesson was the need for comprehensive stakeholder mapping to improve the success of launch efforts. The operationalization of this and other priority items was discussed during the second meeting held in February 2024.

#### Capture success

What success is and how it will be measured will be determined by the FSTWG.

## Discussion

FSTWGs are forums increasingly used in resource-constrained settings for coordinating multisectoral efforts to strengthen food safety control. However, this increased demand is in contrast with the limited discussions on the essential attributes of such coordination mechanisms [32]. This work contributes to the nascent body of literature examining how food safety coordination mechanisms work and how they can be enhanced via integration into existing governmental structures such as One Health platforms [26, 33, 34].

This study examined the evolution of FSTWGs with a long-standing presence in Vietnam and newly established ones in Ethiopia, providing key insights for governments interested in creating FSTWGs. A key takeaway from our experience is that support – technical and financial – by development partners was essential for initiating working groups. Governments may see value in these groups but fail to implement them, likely due to resource constraints. For instance, in Ethiopia, establishing a FSTWG has been within the mandate of NOHSC since inception; however, it was not until the intervention of a development partner (ILRI) that progress was made. Other working groups under NOHSC were similarly initiated with the support of development partners.

Additionally, a major hurdle hindering the effectiveness of FSTWGs over time is the lack of legal frameworks to ensure government commitment, requiring urgent attention. Incorporating these groups within existing government structures from their inception could bolster active engagement and ensure their sustainability in the long run. Additionally, continuing to document the successes of FSTWGs is important for conveying the value-added to stakeholders, such as the extent to which multisectoral coordination informed food safety policies and practices. Furthermore, development partners need to foster ownership of national actors in developing and operationalizing FSTWGs to tackle their food safety issues.

We note a couple of limitations of our study. Firstly, our study relied on the viewpoints of a single founding organization, ILRI, engaged in the creation of FSTWGs, although some co-authors also have affiliations with national institutions, thus adding government perspectives as well. Future studies should explore the experiences of other key organizations and a more extensive membership base. Secondly, our study focused on how FSTWGs work, with only a brief exploration of their outcomes. Conducting process evaluations would help to generate insights to improve their functionality. Furthermore, outcome evaluations could offer deeper insights into the benefits of FSTWGs. Despite these limitations, this research enhanced our understanding of mechanisms that facilitate food safety coordination in LMICs.

## Conclusion

This case study provided a deep dive into the development of FSTWGs operating under a One Health framework in Vietnam and Ethiopia. Despite the two cases having different starting points, similarities were identified in how multisectoral collaborations were established and supported, highlighting the role of development partners in initially championing the process. It also illustrated the different challenges that may be encountered along the way, such as the lack of legal frameworks to secure commitment from governments. Integrating FSTWGs into national processes – such as One Health platforms – could help ensure evidence remains not only with development partners and researchers but is translated into policy and practice to advance food safety. In Vietnam, this integration was achieved through re-structuring of an existing group, whereas in Ethiopia, a completely new group was established. For governments in other LMICs countries looking for insights into setting up technical working groups, our recommendation is to follow the path of least resistance, whether that means adapting an existing group or creating a new one, as both approaches worked well in our experience. As FSTWGs continue to solidify their presence within national One Health mechanisms, future studies are important for understanding how their impact could be amplified when integrated within legal frameworks.

## Data Availability

Not applicable.
